# Anthocyanin biosynthetic pathway switched by metalloregulator PbrR to enable a biosensor for the detection of lead toxicity

**DOI:** 10.3389/fmicb.2022.975421

**Published:** 2022-10-04

**Authors:** Yan Guo, Zhen-lie Huang, De-long Zhu, Shun-yu Hu, Han Li, Chang-ye Hui

**Affiliations:** ^1^National Key Clinical Specialty of Occupational Diseases, Shenzhen Prevention and Treatment Center for Occupational Diseases, Shenzhen, China; ^2^Department of Toxicology, School of Public Health, Southern Medical University, Guangzhou, China; ^3^School of Public Health, Guangdong Medical University, Dongguan, China; ^4^Department of Pathology and Toxicology, Shenzhen Prevention and Treatment Center for Occupational Diseases, Shenzhen, China; ^5^College of Lab Medicine, Hebei North University, Zhangjiakou, China

**Keywords:** bacterial biosensor, anthocyanin biosynthesis, environmental lead, bioavailability, ecotoxic effect

## Abstract

Environmental lead pollution mainly caused by previous anthropogenic activities continuously threatens human health. The determination of bioavailable lead is of great significance to predict its ecological risk. Bacterial biosensors using visual pigments as output signals have been demonstrated to have great potential in developing minimal-equipment biosensors for environmental pollutant detection. In this study, the biosynthesis pathway of anthocyanin was heterogeneously reconstructed under the control of the PbrR-based Pb(II) sensory element in *Escherichia coli*. The resultant metabolic engineered biosensor with colored anthocyanin derivatives as the visual signal selectively responded to concentrations as low as 0.012 μM Pb(II), which is lower than the detection limit of traditional fluorescent protein-based biosensors. A good linear dose–response pattern in a wide Pb(II) concentration range (0.012–3.125 μM) was observed. The color deepening of culture was recognized to the naked eye in Pb(II) concentrations ranging from 0 to 200 μM. Importantly, the response of metabolic engineered biosensors toward Pb(II) was not significantly interfered with by organic and inorganic ingredients in environmental water samples. Our findings show that the metabolic engineering of natural colorants has great potential in developing visual, sensitive, and low-cost bacterial biosensors for the detection and determination of pollutant heavy metals.

## Introduction

Lead (Pb), one of the toxic heavy metals, is a globally prevalent inorganic pollutant that persistently threatens human health (Pourrut et al., 2011). Various instrumental methods such as graphite furnace atomic absorption spectrometry (GFAAS) and inductively coupled plasma mass spectrometry (ICP-MS) are widely used to monitor environmental heavy metals including Pb (USEPA, 2016). However, the information about its bioavailability and ecotoxicity is usually missing in these applications, which is of predictive value for the assessment of its health risk (Hui et al., 2021b).

To address the shortcomings of traditional instrumental methods in determining the bioavailable, bioaccessible, and toxic fractions of heavy metals, some biological devices, especially whole-cell biosensors, have been continuously developed (Kim et al., 2018; Gupta et al., 2019). Based on Pb(II)-responsive metalloregulator PbrR, programmed bacteria could selectively respond to bioavailable Pb(II) with fluorescent proteins and beta-galactosidase as the output signals (Wei et al., 2014; Bereza-Malcolm et al., 2016; Guo et al., 2019; Hui et al., 2020b). However, these whole-cell biosensors failed in the field detection of Pb(II) because the readout of fluorescent and enzymatic signals is highly dependent on the complex instruments. Employment of visual signals has provided an alternative to developing mini-equipment bacterial biosensors responsive to heavy metal pollutions such as Cu(II) (Chen et al., 2017), Cd(II) (Hui et al., 2022a,c), and Hg(II) (Guo et al., 2021a). These pigment-based biosensors have shown more excellent biosensing characteristics than traditional reporters-based biosensors for Cd(II) and Hg(II; Zhang et al., 2021; Guo et al., 2021b; Hui et al., 2021c, 2022b). In our previous studies, bacterial biosensors using blue indigoidine (Hui et al., 2021a), navy violacein (Hui et al., 2020a), and purple deoxyviolacein (Hui et al., 2022d) as the biosensing signals have been successfully developed to detect bioavailable Pb(II). The detection limits of three biosensors had been demonstrated to be lower than the criteria for maximum concentration (CMC) for Pb in freshwater (0.31 μM) recommended by the United States Environmental Protection Agency (USEPA) (USEPA, 2016). The detection limit of the whole-cell biosensors using indigoidine and deoxyviolacein as the visual signals was even lower than the national primary drinking water standard (0.072 μM) recommended by USEPA (USEPA, 2009). Due to the rapid development of metabolic engineering and synthetic biology, heterologous biosynthesis of rainbow colorants had been realized in programmed bacteria (Yang et al., 2020, 2021). The development of novel bacterial biosensors using colorful pigments as visual reporters would provide us with more biological devices for the sensitive detection of toxic heavy metals in different applications.

Anthocyanins are red, purple, or blue water-soluble pigments found in terrestrial plants, which belong to the family of polyphenolic compounds (Yan et al., 2005). Their anti-oxidative, anti-cancer, anti-inflammatory, and cardioprotective properties have attracted much attention to the reconstruction of their biosynthetic metabolic pathways (Yan et al., 2008). In the study, two key biosynthetic enzymes were employed to reconstruct the biosynthetic metabolic pathway flux toward anthocyanin, which is designed to be triggered by Pb(II)-responsive metalloregulator PbrR. Engineered bacterial biosensors selectively responded to bioavailable Pb(II) at the nanomolar level by secreting colored anthocyanidin derivatives (CACD) with maximum absorption at 428 nm into the culture. Importantly, the CACD-based whole-cell biosensor was validated in detecting bioavailable and toxic Pb(II) existing in both experimental and environmental samples. Compared with traditional fluorescent reporters-based or enzymatic reporters-based whole-cell biosensors, metabolic engineering of natural colorants was shown to have the potential to be employed as the visual reporter to develop a low-cost, mini-equipment biosensor for heavy metals. The sole cost of culture medium and consumable material is negligible, and only an incubator and a microplate reader are the necessary instruments.

## Materials and methods

### Bacterial strains, plasmids, and agents

The vectors, bacterial host, and engineered bacterium involved in this study are listed in Supplementary Table S1. *Escherichia coli* (*E. coli*) TOP10 was used as the bacterial host for the gene cloning and biosensing tests. The engineered bacterium was cultured at 37°C in Luria-Bertani (LB) broth containing 1% tryptone, 0.5% yeast extract, and 1% sodium chloride supplemented with 50 μg/ml ampicillin. Reagents for molecular cloning were obtained from Sangon Biotech (Shanghai, China). The recombinant constructs were all verified by DNA sequencing (Sangon Biotech). (+)-catechin was purchased from Sigma-Aldrich (St Louis, MO, United States), dissolved in ethanol to achieve a final concentration of 125 mM, and stored in the dark at 4°C. Cadmium chloride, lead nitrate, zinc sulfate, and mercuric chloride are of analytical grade and purchased from Sigma-Aldrich (St Louis, MO, United States). Stock solutions of metal salts were freshly prepared and filtered through a 0.22 μM filter before the experiments.

### Genetic assembly of pigment-based biosensor

To reconstruct the anthocyanin biosynthetic module, a bicistronic genetic fragment encoding two open reading frames (ORFs) designated as 3-*O*-glycosyltransferase originating from *Arabidopsis thaliana* (Genbank: AY072325) and anthocyanidin synthase originating from *Petunia hybrid* (Genbank: P51092) was artificially synthesized by Sangon Biotech according to the *E. coli* codon preference (Supplementary Figure S1). The synthetic anthocyanin biosynthetic cassette was PCR amplified from the vector pT-3GT-ANS and inserted into the *Nde*I and *Sac*I sites of pPpbr-vio to generate pPb-CACD, which is designed as a bioavailable Pb(II) biosensing construct with the CACD as the output signals. The resultant pPb-CACD was used for transformation into *E. coli* TOP10 competent cells. Whole-cell biosensors TOP10/pPb-CACD were selected on LB agar plates containing 50 μg/ml ampicillin.

### Characterization of Pb(II)-driven color signal from the pigment-based biosensor

To obtain the Pb(II)-induced water-soluble pigment, overnight LB culture of TOP10/pPb-CACD was diluted 1:100 in fresh LB medium supplemented with or without 1 mM catechin. Engineered TOP10/pPb-CACD was then induced with 50 μM Pb(II) overnight (about 15 h) at 37°C with shaking at 250 rpm. The cell-free culture supernatants were prepared by centrifugation at 8000 g for 1 min, pipetted into a 96-well microplate, and scanned in a microplate reader (BioTek Epoch, United States). A 300–750 nm scanning wavelength range with an interval of 2 nm was set.

### Detection selectivity assay

To evaluate the detection selectivity of engineered bacterium, TOP10/pPb-CACD preserved in 20% glycerol was diluted 1:100 in fresh LB medium supplemented with 1 mM catechin. Stock solutions of Cd(II), Pb(II), Zn(II), and Hg(II) were then added to the cultures at a final concentration of 0, 1.25, 2.5, 5, 10, and 20 μM, followed by incubation overnight at 37°C with shaking at 250 rpm. Aliquots of 100 μl culture and cell-free supernatant were pipetted into a 96-well microplate and determined at 600 nm and 428 nm for bacterial density and color signal, respectively.

### Detection sensitivity assay

To study the dose–response relationship of engineered bacterium toward increased concentrations of Pb(II), TOP10/pPb-CACD preserved in 20% glycerol was diluted 1:100 in fresh LB medium supplemented with 1 mM catechin, and exposed to 200, 100, 50, 25, 12.5, 6.25, 3.125, 1.56, 0.78, 0.39, 0.195, 0.098, 0.049, 0.024, 0.012, 0.006, and 0 μM Pb(II) using a double dilution method as described previously (Hui et al., 2022b). After incubation overnight at 37°C with shaking at 250 rpm, bacterial density and color signal were measured as described above.

### Detection of bioavailable Pb(II) in environmental water samples

To validate the capability of an engineered bacterium to sense the soluble, bioaccessible, and bioavailable Pb(II) in environmental water samples, recombinant TOP10/pPb-CACD preserved in 20% glycerol was diluted 1:100 in fresh LB medium prepared with purified water, tap water from our laboratory, and surface water from two downtown parks as described previously (Hui et al., 2022a,c,d). The cultures were supplemented with 1 mM catechin, spiked with 25, 12.5, 6.25, 3.125, 1.56, 0.78, 0.39, and 0 μM Pb(II) in a double dilution method, and then followed by incubation overnight at 37°C with shaking at 250 rpm, bacterial density and color signal were measured as described above.

## Results and discussion

### Design of Pb(II)-responsive bacterial biosensor based on the anthocyanin biosynthetic pathway

**Figure 1:**
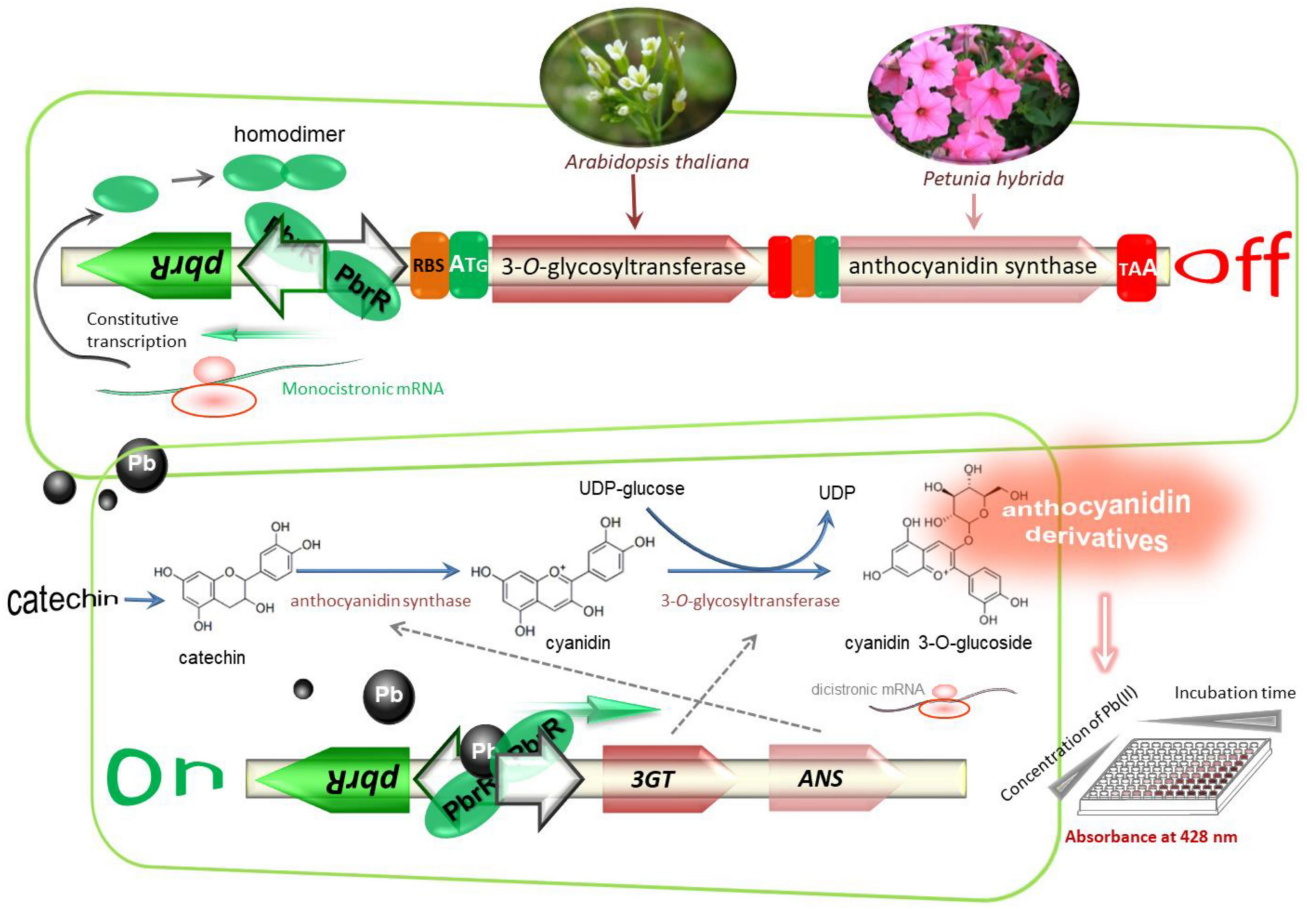
Reconstruction of the anthocyanidin biosynthetic pathway under the control of Pb(II)-responsive genetic element. The anthocyanidin biosynthesis genes cluster was artificially synthesized and inserted downstream of the divergent *pbr* promote to assemble Pb(II) biosensing construct. Anthocyanidin synthase originating from *Petunia hybrida* and 3-*O*-glycosyltransferase originating from *Arabidopsis thaliana* are expected to be transcribed and translated as a bicistronic unit when the metabolically engineered bacterium is exposed to bioavailable Pb(II). The anthocyanidin cyanidin 3-O-Glucoside is biosynthesized by catalyzing the production of cyanidin from the substrate catechin by ANS and transferring a glucose group to cyanidin to yield C3G by 3GT. Water-soluble CACD will be secreted by biosensor cells and quantitatively measured at 428 nm in a 96-well microplate.

The construction scheme and molecular mechanism of the whole-cell biosensor based on the anthocyanidin biosynthesis which is switched by bioavailable Pb(II) are summarized in Figure 1. Anthocyanidins, as valuable natural colorants, have always attracted intense attention due to their beneficial health effects (Yan et al., 2008). Their biosynthesis pathways have been extensively investigated (Yang et al., 2021). Two key enzymes including anthocyanidin synthase (ANS) and 3-*O*-glycosyltransferase (3GT) are essential in the biosynthesis of the anthocyanin cyanidin 3-O-glucoside (C3G; Yan et al., 2005). The improved production of C3G has been achieved by using a bicistronic 3GT-ANS expression cassette (Lim et al., 2015). The same bicistronic genetic module was employed as a reporter module under the control of the Pb(II) sensory element in the present study. Upon exposure to bioavailable Pb(II), metabolic engineered bacterial biosensors are expected to secrete water-soluble colorants into the culture supernatant by triggering the transcription of the bicistronic pigment biosynthesis genes (Figure 1).

**Figure 2:**
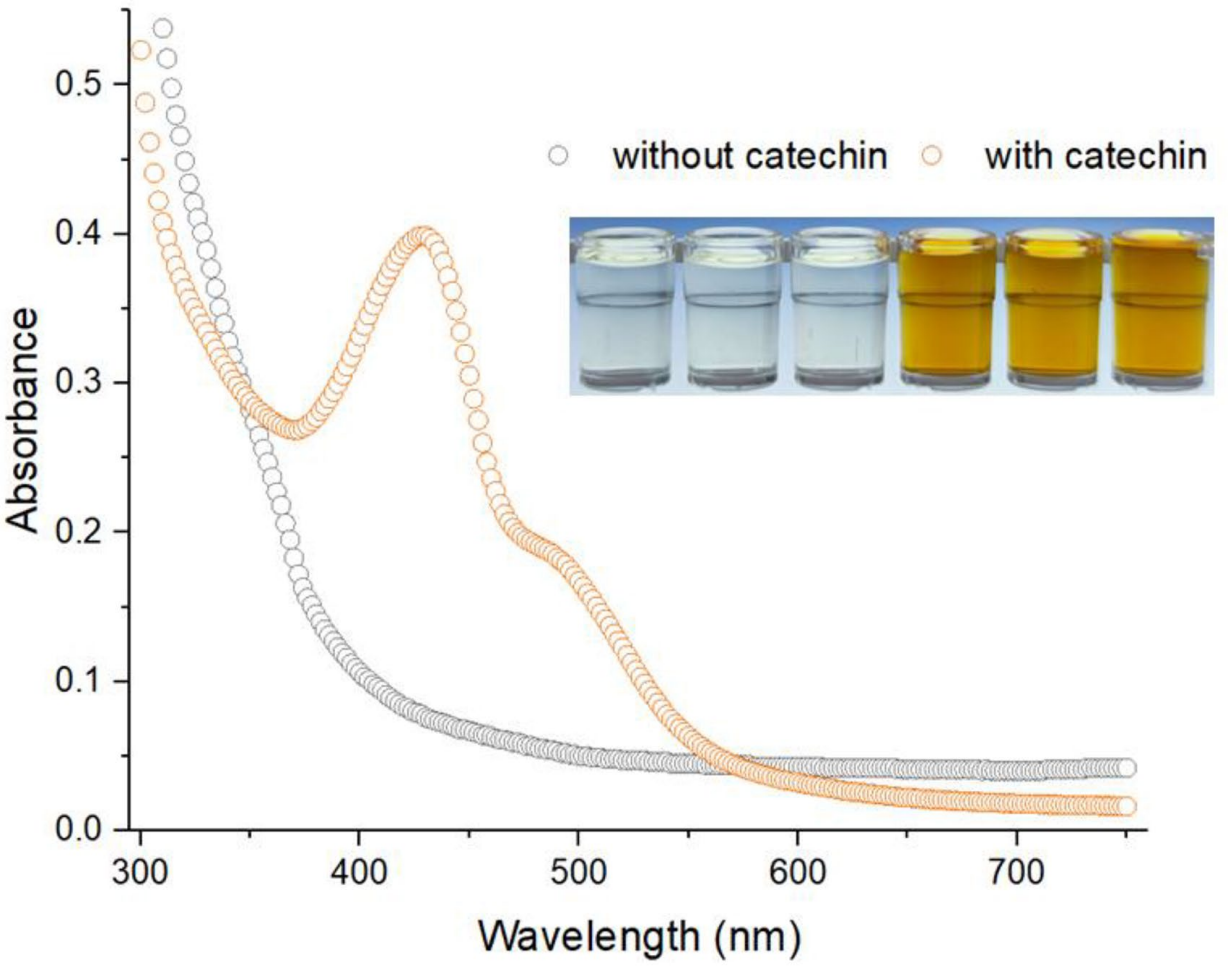
The visible absorption spectrum of water-soluble CACD generated from Pb(II)-induced TOP10/pPb-CACD. The inset shows the cell-free culture supernatants of 50 mM Pb(II)-induced TOP10/pPb-CACD supplemented with or without 1 mM catechin. The maximum absorption peak of secreted CACD is located at around 428 nm. Data are representative of three independent tests with similar results.

Anthocyanins are relatively stable under acidic conditions and rapidly break down under neutral conditions (Tsuda et al., 2005). Significant degradation of C3G was previously observed at pH 7.0. However, C3G was stable at pH 5.0 or lower (Yan et al., 2008). The pH value of the culture was maintained at about 7.0 in the present study, which was suitable for the growth of a bacterial biosensor. However, the product C3G was demonstrated to degrade severely in the current condition. As shown in Figure 2, the visible absorption spectra of 50 mM Pb(II)-induced culture supernatant containing CACD showed maximum absorption at 428 nm, which was different from the maximum absorption of the red-colored C3G at 512 nm reported previously (Lim et al., 2015). HPLC was usually used for product analysis of fermentation samples for pigment production. Residual anthocyanidin could be conveniently identified by retention times and UV absorption spectra (Yan et al., 2008). However, there are few studies about the identification of its degradation products. We infer that the anthocyanin is seriously degraded through the change of color and the migration of maximum absorption wavelength. This conclusion needs to be further identified by reliable measurements such as HPLC-MS in future studies. Thus, the CACD-derived signal was determined at 428 nm instead of 512 nm in the following study.

The cascade amplification effect of enzymatic reporters made whole-cell biosensors using enzyme-based reporters such as luciferase and beta-galactosidase are more sensitive than fluorescent proteins-based biosensors (Hui et al., 2021b). Because a small amount of expressed enzymes can overproduce visible amounts of pigment (McNerney et al., 2019), the continuous biosynthesis of reporter pigments with the extension of heavy metals induction time was commonly observed in previously developed pigment-based biosensors (Guo et al., 2021a; Hui et al., 2022a,d). The continuous production accompanied by the degradation of C3G was also observed in Pb(II)-induced TOP10/pPb-CACD grown in a regular LB medium (at pH 7.0). However, the dose–response relation became less obvious to the naked eye with the extension of induction time (Supplementary Figure S2A). Furthermore, continuous deepening of the color was also observed when Pb(II)-induced culture was placed at 37°C (Supplementary Figure S2B). To exclude the influence of the biosensor cells containing anthocyanin biosynthetic enzymes, the stability of cell-free culture supernatant was investigated at both acidic and neutral pH (Supplementary Figure S3). No color change was observed in acidic culture supernatant and the result demonstrated that anthocyanin compounds are stable at acidic pH which is consistent with previous reports (Springob et al., 2003; Yan et al., 2008). Taking this into consideration, a constant incubation time (15 h) followed by an immediate measurement was used in the following study to obtain the colorimetric signal of CACD.

To avoid spontaneous chemical degradation of red C3G in a fermentation culture, a two-step fermentation strategy is deserved to be developed in the future study. High biomass was first obtained by growing biosensor cells in a regular medium at neutral pH and then exposed to toxic Pb(II) in a modified medium at acidic pH to reduce the degradation of reporter C3G.

### Selective response of the metabolic engineered bacterial biosensor

**Figure 3:**
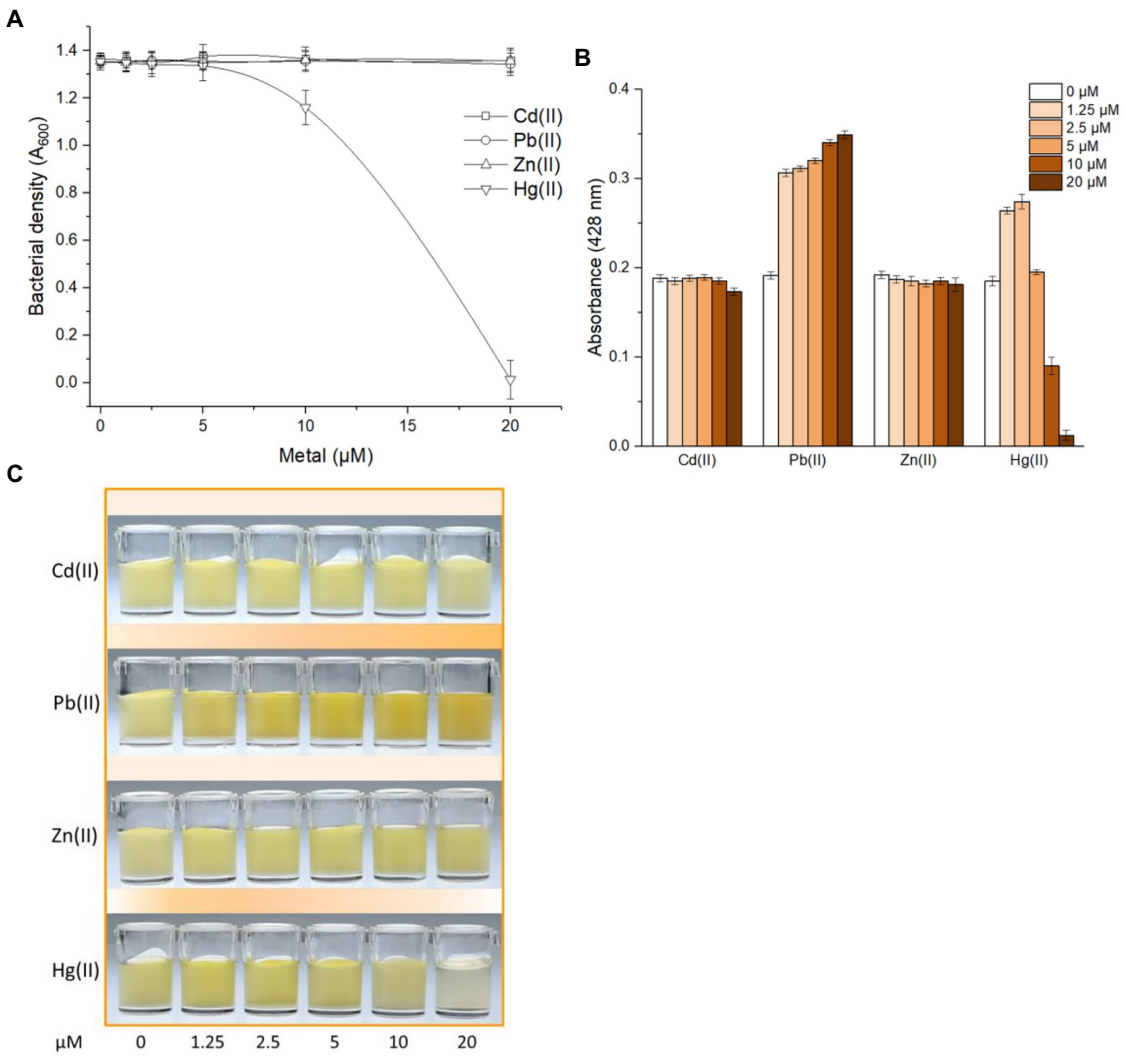
Differential responses of TOP10/pPb-CACD toward various metal ions. TOP10/pPb-CACD was inoculated into fresh LB medium supplemented with 1 mM catechin and exposed to increased concentrations of Cd(II), Pb(II), Zn(II), or Hg(II). After culture at 37°C overnight, bacterial cell densities **(A)** and pigment signal **(B)** was measured at 600 nm and 428 nm, respectively. Data represent the average of three independent assays with similar results and error bars represent standard deviations. Data shown are mean ± SD (*n* = 3). A representative picture of induced cultures **(C)** was chosen from three independent tests with similar results.

MerR-like transcriptional regulators do not distinguish chemically related group 12 metals well (Caguiat et al., 1999; Hakkila et al., 2011). The weak nonspecific responses of MerR-family metalloregulators toward metals especially group 12 members were found in Pb(II)-responsive PbrR (Bereza-Malcolm et al., 2016), Cd(II)-responsive CadR (Hui et al., 2022b), Cd(II)-responsive CadC (Hui et al., 2021c), Zn(II)-responsive ZntR (Kang et al., 2018), and so on. A slight cross-response of metabolic engineered biosensor toward Hg(II) was found in this study. No significant growth inhibition led by toxic Hg(II) was observed at 5 μM or less (Figure 3A) and an enhanced response to 1.25 and 2.5 μM Hg(II) was observed in Figure 3B. Importantly, a dose-dependent response toward Pb(II) was preliminary verified at concentrations of Pb(II) ranging from 0 to 20 μM (Figure 3B). Furthermore, the color of Pb(II)-induced cultures gradually deepened which was recognizable to the naked eye (Figure 3C).

The response selectivity of the bacterial biosensor using PbrR as the sensory element was demonstrated to be determined by the inherent characteristics of native PbrR (Chen et al., 2005). However, the optimizations of gene circuits (Cai et al., 2018; Jia et al., 2018), the directed evolution of metalloregulators (Cai et al., 2022), and the combined use of several metal sensory elements (Hui et al., 2022b) have been demonstrated to improve the performance of a whole-cell biosensor toward chemically-similar metals. Although the novel pigment-based actuator developed in this study did not contribute to improving the metal selectivity of whole-cell biosensors, the combination of pigment reporter with a modified metal sensory module is expected to improve the biosensing properties including metal selectivity in the future study.

### Response properties of metabolic engineered bacterial biosensor toward Pb(II)

**Figure 4:**
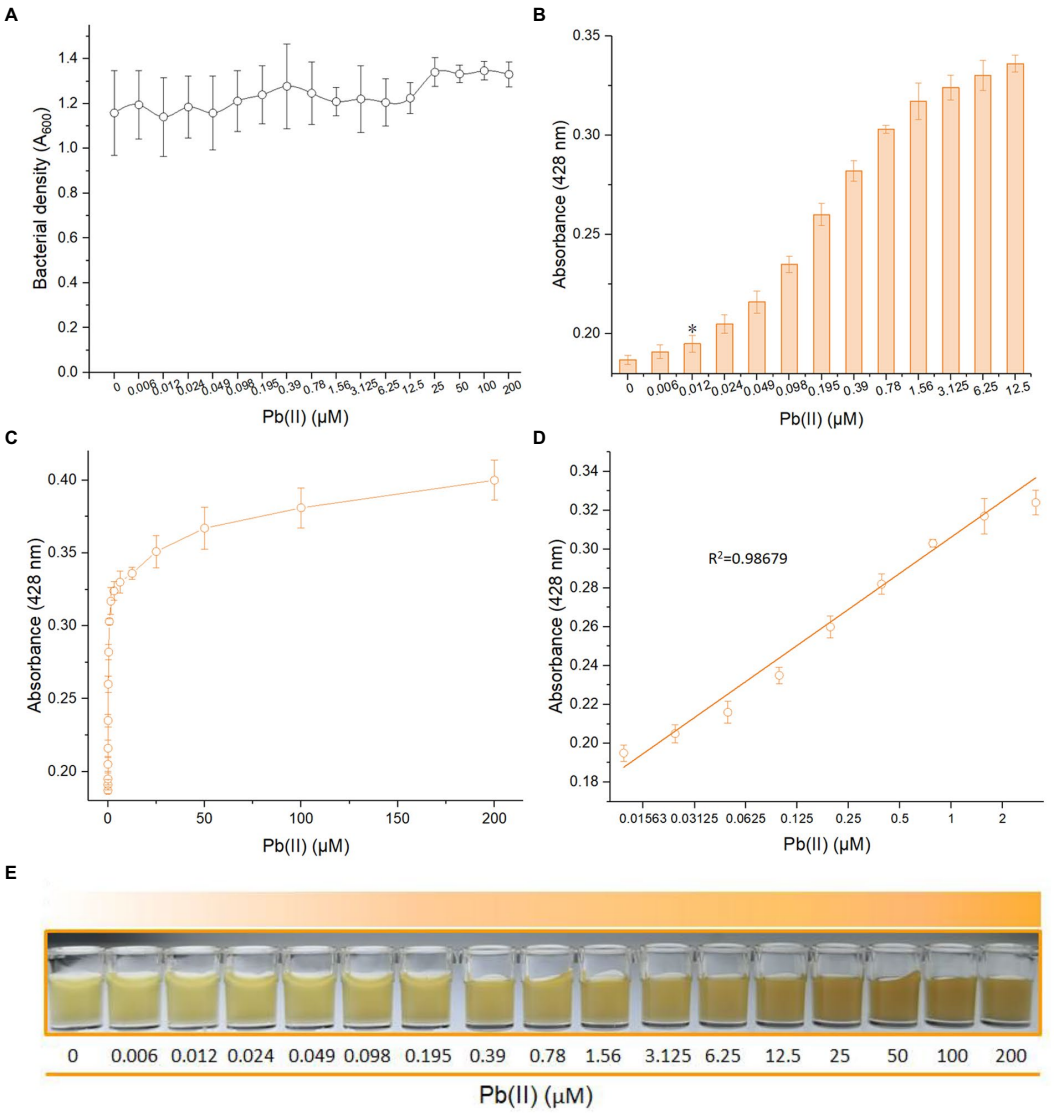
Response of TOP10/pPb-CACD to increased concentrations of Pb(II). TOP10/pPb-CACD was inoculated into fresh LB medium supplemented with 1 mM catechin and induced with increased concentrations of Pb(II) at 37°C overnight. The cell-free culture supernatants containing CACD were prepared and detected by visible light absorbance at 428 nm. **(A)** Bacterial densities of TOP10/pPb-CACD exposed to different concentrations of Pb(II). **(B)** The detection sensitivity of TOP10/pPb-CACD. Data represent the average of three independent assays with similar results. Error bars represent standard deviations. The asterisk represents the limit of detection, which is adopted as the lowest concentration of Pb(II) that triggered a significant increase of absorbance value at 428 nm (background +3 × SD). **(C)** The dose–response curve with Pb(II) concentration ranges from 0 to 200 μM. **(D)** Regression analysis of the relationship of pigment-based signal and the Pb(II) concentrations ranging from 0.012 to 3.125 μM. The *x*-axis displays the Pb(II) concentration on the log_2_ scale. Data shown are mean ± SD (*n* =3). **(E)** A representative picture of the cultures induced with increased concentrations of Pb(II).

To further study the dose–response relationship, metabolic engineered TOP10/pPb-CACD was exposed to systematically varied concentrations of Pb(II). Due to the low cytotoxic activity of Pb(II), the bacterial concentration did not show a decreasing trend (Figure 4A). Recombinant TOP10/pPb-CACD was demonstrated to respond to concentrations as low as 0.012 μM Pb(II) using a colorimetric method (Figure 4B). The detection limit of this biosensor is lower than the CMC for Pb in freshwater (0.31 μM) and the primary drinking water standard (0.072 μM) recommended by USEPA (USEPA, 2009, 2016). The result showed that this metabolically engineered biosensor was expected to detect toxic Pb(II) in the ecosystem to early warn acute toxicity in aquatic organisms. However, the detection limit of the PbrR-based bacterial biosensor using the fluorescent protein as a reporter was increased to 0.97 μM (Bereza-Malcolm et al., 2016).

The whole dose–response of TOP10/pPb-CACD toward Pb(II) was shown in Figure 4C, which was similar to that of the previously constructed PbrR-based bacterial biosensor with the blue pigment indigoidine as the output signal (Hui et al., 2021a). The CACD-derived signal was significantly increased from 0 to 12.5 μM and gradually stabilized from 25 to 200 μM (Figure 4C). The constant expression of PbrR is attributed to the regulation of the natural *pbr* promoter (Borremans et al., 2001). Before the metal binding sites of PbrR were saturated with Pb(II), the transcription of pigment biosynthetic enzymes would continuously improve with the increased Pb(II) concentration, and it led to the increased pigment signal until the metal binding sites of PbrR were saturated with excess Pb(II) (Hui et al., 2022d). Interestingly, the Pb(II) concentration (log_2_ scale) and the absorbance of CACD could be well fitted by a linear regression relation within a wide Pb(II) concentration range between 0.012 and 3.125 μM (Figure 4D). Importantly, the gradual color deepening of Pb(II)-induced culture was easily recognized by the naked eye independent of any instruments (Figure 4E).

### The performance of metabolic engineered bacterial biosensor in monitoring bioavailable Pb(II) in environmental samples

**Figure 5:**
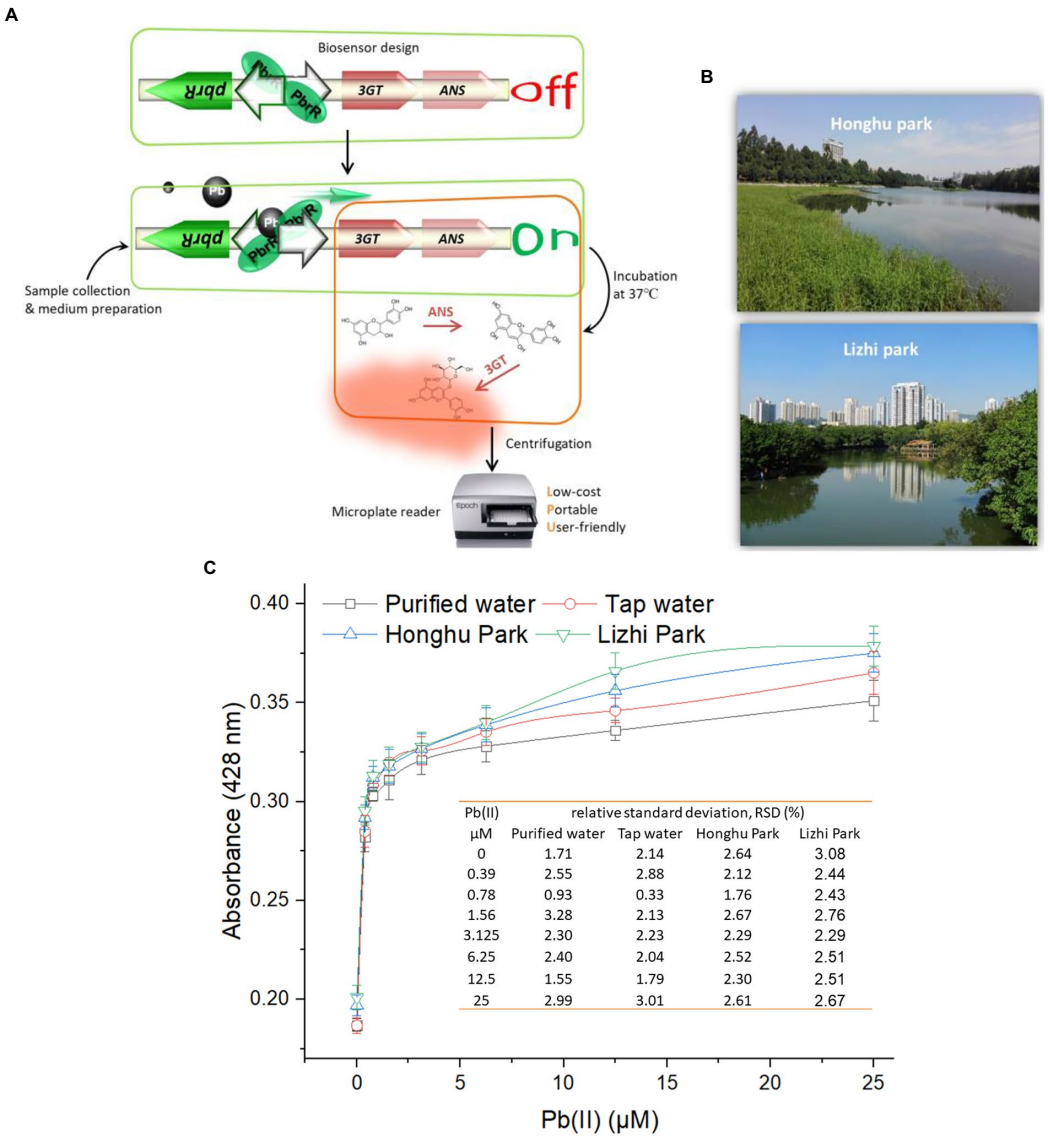
Detection of bioavailable Pb(II) in environmental samples using TOP10/pPb-CACD. **(A)** The detailed protocol for the field measurement. The capability of TOP10/pPb-CACD was validated in determining bioaccessible Pb(II) in environmental samples using a three-step colorimetric method. Surface water was collected from two downtown parks in Shenzhen city, South China **(B)**. TOP10/pPb-CACD was inoculated into fresh LB medium prepared with four different water samples and supplemented with 1 mM catechin. Different concentrations of Pb(II) were spiked into the culture system. After culturing at 37°C overnight, the cell-free culture supernatants containing CACD were prepared and determined at 428 nm **(C)**. Data shown are mean ± SD (*n* = 3). The inset table shows the relative standard deviation (RSD) of pigment derived signal.

A simple protocol for the determination of bioavailable Pb(II) using this metabolically engineered biosensor was drawn up based on the above findings (Figure 5A). The culture system is first prepared using up to 90% of lead polluted environmental water samples to be tested, inoculation with the bacterial biosensor, followed by incubation at 37°C for 15 h in a constant temperature shaker. The CACD-derived signal is then measured at 428 nm using a microplate reader after a simple centrifugation step. The whole test can be finished within one day.

The capability of a novel biosensor in detecting toxic heavy metals in environmental water samples is important for evaluating its potential application in the real world. Environmental surface water was collected from two downtown parks and the sampling sites were shown in Figure 5B. Total Pb in collected tap water and two kinds of environmental surface water was detected using the ICP-MS and demonstrated to be below the detection limit (0.145 nM). Artificially contaminated environmental samples were usually used when no heavy metals polluted environmental sample was available (Kumar et al., 2017; Hui et al., 2022c). In this study, freshly prepared culture systems using different water samples were artificially contaminated by spiking with different concentrations of soluble Pb(II), inoculated with TOP10/pPb-CACD, and incubated overnight at 37°C. The four culture systems were maintained at about neutral pH during the whole process. As expected, the response patterns showed a striking similarity in the dose–response curves among all four groups, and the relative standard deviations were all below 3.3 (Figure 5C).

Many factors including the pH value, hardness, and dissolved organic matter (DOM) in the aquatic system can affect the speciation of heavy metals, which is vital for their bioavailability and ecotoxic effects (Ryan et al., 2009). However, the inorganic ions and DOM existing in the natural aquatic system only exerted a slight influence on the Pb(II)-responsive property of TOP10/pPb-CACD, which is similar to previously developed pigment-based biosensors (Guo et al., 2021a; Hui et al., 2022a,c).

Different from dithizone colorimetry, this biosensing technique has obvious advantages in sensitivity and specificity. Compared with high-precision instruments such as atomic absorption spectrometry and ICP-MS, low selectivity, and low sensitivity can also be improved by modifying Pb(II) sensory module and genetic circuits in the future study. Importantly, monitoring of bioaccessible, bioavailable, and toxic Pb(II) is essential in predicting its ecotoxicity and health risk, which makes the whole-cell biosensor become a powerful supplement to above traditional methods. In summary, our study shows that a metabolic engineered bacterial biosensor has the potential to become a low-cost, portable, and user-friendly biological device for detecting environmental Pb(II).

## Conclusion

Limited attempts have been made to assemble heavy metal whole-cell biosensors using the natural pigment synthesis pathway. In this study, the Pb(II)-responsive metabolic flow toward anthocyanin enabled a minimal-equipment whole-cell biosensor. The resultant CACD could be quantitatively determined at 428 nm in a colorimetric method. The metabolically engineered biosensor selectively responded to Pb(II), the detection limit was 0.012 μM, and the quantitative detection range was 0.012–3.125 μM. The colorimetric bacterial biosensor was validated in detecting bioavailable Pb(II) in artificially polluted environmental water samples. Our study shows that bacterial biosensor modified by metabolic engineering is promising for environmental monitoring of heavy metal pollution sensitively and visually.

## Data availability statement

The original contributions presented in the study are included in the article/Supplementary material, further inquiries can be directed to the corresponding author.

## Author contributions

C-yH and YG designed the experimental protocol. C-yH drafted the manuscript. D-lZ, S-yH, and HL carried out the majority of the study. YG and Z-lH analyzed the data. All authors contributed to the article and approved the submitted version.

## Funding

This work was supported by the National Natural Science Foundation of China (82073517), the Natural Science Foundation of Guangdong Province (2019A1515011989 and 2021A1515012472), the Science and Technology Program of Shenzhen (JCYJ20180306170237563, JCYJ20190808175205480, and KCXFZ20201221173602007), Shenzhen Key Medical Discipline Construction Fund (SZXK068), and Shenzhen Fund for Guangdong Provincial High-level Clinical Key Specialties (SZGSP015).

## Conflict of interest

The authors declare that the research was conducted in the absence of any commercial or financial relationships that could be construed as a potential conflict of interest.

## Publisher’s note

All claims expressed in this article are solely those of the authors and do not necessarily represent those of their affiliated organizations, or those of the publisher, the editors and the reviewers. Any product that may be evaluated in this article, or claim that may be made by its manufacturer, is not guaranteed or endorsed by the publisher.
