# Supplementary material for: Anthocyanin biosynthetic pathway switched by metalloregulator PbrR to enable a biosensor for the detection of lead toxicity
Source: Front Microbiol. 2022 Oct 4;13:975421. doi: 10.3389/fmicb.2022.975421 (PMC9577363; doi:10.3389/fmicb.2022.975421)
Supplement: Supplementary file 1 [file Data_Sheet_1.docx]

**Supplementary data**

**Anthocyanin biosynthetic pathway switched by metalloregulator PbrR to enable a biosensor for the detection of lead toxicity**

Yan Guo^1^, Zhen-lie Huang^2^, De-long Zhu^3,5^, Shun-yu Hu^2,1^, Han Li ^4,5^, Chang-ye Hui^5*^

^1^ National Key Clinical Specialty of Occupational Diseases, Shenzhen Prevention and Treatment Center for Occupational Diseases, Shenzhen, China

^2^ Department of Toxicology, School of Public Health, Southern Medical University, Guangzhou, China.

^3^ School of Public Health, Guangdong Medical University, Dongguan, China.

^4^ College of Lab Medicine, Hebei North University, Zhangjiakou, China

^5^ Department of Pathology & Toxicology, Shenzhen Prevention and Treatment Center for Occupational Diseases, Shenzhen, China

*Correspondence author

*E-mail addresses*: hcy_sypu@hotmail.com

**Table. S1** Bacterial strains and plasmids used in this study

| **Strains and plasmids** | **Genotypes or description** | **Reference** |
| --- | --- | --- |
| ***E. coli* strains** |  |  |
| TOP10 | F^-^ Φ80*lac*ZΔM15 Δ*lac*X74 *rec*A1 | Invitrogen |
| TOP10/pPb-CACD | *E. coli* TOP10 harboring Pb(II) biosensing construct pPb-CACD | This study |
| **Plasmids** |  |  |
| pPpbr-vio | pET-21a derivative containing the violacein biosynthetic gene cluster under the control of the Pb(II) sensory module | [^1^](#_ENREF_1) |
| pT-3GT-ANS | T vector carrying *A. thaliana 3GT and P. hybrid ANS* bicistronic unit | This study |
| pPb-CACD | pPpbr-vio derivative containing the anthocyanin biosynthetic module under the control of the Pb(II) sensory module | This study |

AGATCTTTACCCAGATGTTTGACTGTTCGTGGCACTTTCACCATGGCAATTGCCCAACCCTTGCAAAATGCCGCACGCCTCTACAGATCGAGAGCCAGAACACTTCTCGCGCAAATCAACCAAGTGCCGTTTTAACTGCAACAGCGCGGACACACGCATTTCCACCTGTTGAATATGGGCCTCCAGCAGCGTGATGACCTCCCCACAGTCCTGCATCGGGTTGTCTCGCAGACCCAGCAATGCGCGAATCTCGCTCAACGTCATGTCGAGCGAACGGCAATGACGGATGAATTGCAAGCGCTCAATGTGCGCCTCACCGTACAACCGAAAGTTGCCACCGCTTCGCGCTGGCTTTGGCAGTAGCCCTTCCTTCTCGTAGTAGCGGATGGTCACGACCTCGCACCCAGAGCGCTTGGCGAGGTCGCCAATTCTGATTTCCAT**GCATCAATCTCCAATTATCACTTGACTCTATAGTGACTATAGAGATTTTAATGGAGGCTGAATAGAAGATTTTCAGGAGTTACTC**TCTAGAAATAATTTTGTTTAACTTTAAG**AAGGAG**ATATACAT**ATG**ATGACCAAACCGTCTGATCCGACCCGTGACTCTCACGTTGCGGTTCTGGCGTTCCCGTTCGGCACCCACGCTGCCCCGCTGCTGACCGTTACCCGTCGCCTGGCGTCCGCCTCTCCGTCTACCGTTTTCAGCTTCTTTAACACCGCGCAGTCTAACTCTAGCCTGTTCAGCTCTGGTGACGAAGCTGATCGTCCGGCTAACATCCGTGTGTATGATATCGCGGACGGCGTGCCGGAAGGCTACGTTTTCTCTGGCCGTCCGCAGGAAGCGATCGAACTGTTTCTGCAAGCGGCGCCGGAAAACTTCCGTCGTGAAATTGCGAAAGCTGAAACCGAAGTTGGCACTGAAGTTAAATGCCTGATGACCGATGCGTTCTTCTGGTTCGCTGCGGATATGGCAACCGAAATCAACGCTTCCTGGATCGCGTTTTGGACCGCGGGTGCTAACTCTCTGAGCGCGCACCTGTATACCGACCTTATCCGTGAAACTATCGGCGTAAAAGAAGTGGGTGAACGCATGGAAGAAACCATCGGCGTGATCTCTGGTATGGAAAAAATCCGTGTTAAAGATACCCCGGAAGGCGTGGTTTTCGGCAACCTGGATAGCGTGTTCAGCAAAATGCTGCACCAGATGGGCCTGGCGCTGCCGCGTGCTACCGCGGTGTTCATCAACAGCTTCGAAGATTTAGATCCGACCCTGACCAACAACCTGCGTTCTCGCTTCAAACGTTACCTGAACATCGGCCCGCTGGGCCTGCTGTCTAGCACCCTGCAACAGCTGGTGCAGGACCCGCATGGTTGCCTGGCGTGGATGGAAAAACGTTCCTCTGGCAGCGTGGCGTACATTTCCTTCGGCACCGTTATGACCCCGCCGCCGGGTGAACTGGCGGCCATTGCAGAAGGCCTGGAATCTAGCAAAGTGCCGTTCGTTTGGTCTCTGAAAGAAAAAAGCCTGGTGCAGCTGCCGAAAGGCTTCCTGGATCGTACCCGTGAACAGGGCATCGTTGTACCGTGGGCGCCGCAGGTTGAACTGCTGAAACACGAAGCGACCGGCGTATTCGTTACCCACTGCGGCTGGAACAGCGTTCTGGAATCCGTTTCCGGTGGCGTTCCGATGATTTGCCGTCCGTTCTTCGGCGATCAGCGTCTGAACGGTCGTGCTGTGGAAGTTGTTTGGGAAATTGGTATGACCATTATCAACGGTGTTTTTACTAAAGACGGTTTCGAAAAATGCCTGGATAAAGTGCTGGTTCAGGACGATGGCAAGAAAATGAAATGCAACGCGAAAAAACTGAAAGAACTGGCGTACGAAGCGGTTAGCTCCAAAGGTCGTTCTTCCGAAAACTTCCGTGGCCTGCTGGACGCAGTTGTTAACATCATC**TAA**TT**AAGGAGG**TAAAAAAA**ATG**ATGGTTAACGCGGTTGTTACCACCCCGAGCCGTGTTGAATCCCTGGCGAAAAGCGGCATCCAGGCGATCCCGAAAGAATACGTTCGTCCGCAGGAAGAACTGAACGGCATCGGTAACATTTTCGAAGAAGAGAAAAAAGATGAAGGTCCGCAGGTTCCGACCATCGATCTGAAAGAAATCGATAGCGAAGATAAAGAAATCCGTGAAAAATGCCACCAGGAACTGAAAAAAGCGGCGATGGAATGGGGCGTTATGCACCTGGTTAACCACGGCATCAGCGATGAACTGATCAACCGTGTTAAAGTGGCGGGTGAAACCTTCTTCGATCAGCCGGTTGAAGAAAAAGAAAAATACGCGAACGATCAGGCTAACGGCAACGTTCAGGGTTACGGTAGCAAACTGGCGAACTCCGCTTGCGGTCAGCTGGAATGGGAAGATTACTTCTTCCACTGCGCGTTCCCGGAAGATAAACGTGATCTGAGCATCTGGCCGAAAAACCCGACCGATTACACCCCGGCGACCAGCGAATACGCGAAACAGATCCGTGCTCTGGCGACCAAAATCCTGACCGTTCTGAGCATCGGCCTGGGCCTGGAAGAAGGCCGTCTGGAAAAAGAAGTGGGTGGTATGGAGGATCTGCTGCTGCAAATGAAAATCAACTACTACCCGAAATGCCCGCAGCCGGAACTGGCGCTGGGTGTGGAAGCTCACACCGACGTTTCTGCGCTGACCTTCATCCTGCACAACATGGTTCCGGGCCTCCAGCTGTTCTACGAAGGTCAGTGGGTTACCGCGAAATGCGTGCCGAACAGCATCATCATGCACATCGGTGATACCATCGAAATCCTGAGCAACGGCAAATACAAATCCATCCTGCACCGTGGCGTTGTTAATAAAGAAAAAGTTCGTATCTCTTGGGCGATTTTCTGTGAACCGCCGAAAGAAAAAATCATCCTGAAACCGCTGCCGGAAACCGTTACTGAAGCTGAACCGCCGCGCTTCCCGCCGCGTACCTTCGCGCAGCACATGGCACACAAACTGTTCCGTAAAGATGATAAAGATGCAGCGGTTGAACACAAAGTTTTCAAAGAAGATGAACTGGATACCGCGGCGGAACACAAAGTTCTGAAAAAAGATAACCAGGACGCGGTTGCGGAGAACAAAGATATCAAAGAAGATGAACAGTGTGGCCCGGCTGAACACAAAGATATTAAAGAAGATGGTCAGGGTGCGGCCGCCGAAAACAAAGTTTTTAAGGAGAATAACCAGGACGTAGCGGCTGAAGAATCTAAA**TAA**GAGCTC


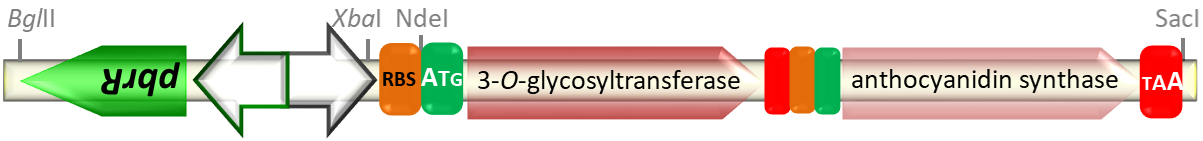


**Fig. S1 The pigment-based Pb(II) biosensing construct used in this study.**

The artificial anthocyanin biosynthetic gene cluster spans 2.7 kilobases and is comprised of *3GT* (1386 bp) and *ANS* (1299 bp), which is inserted downstream of the Pb(II) sensory module containing the *pbrR* gene and the divergent *pbr* promoter.


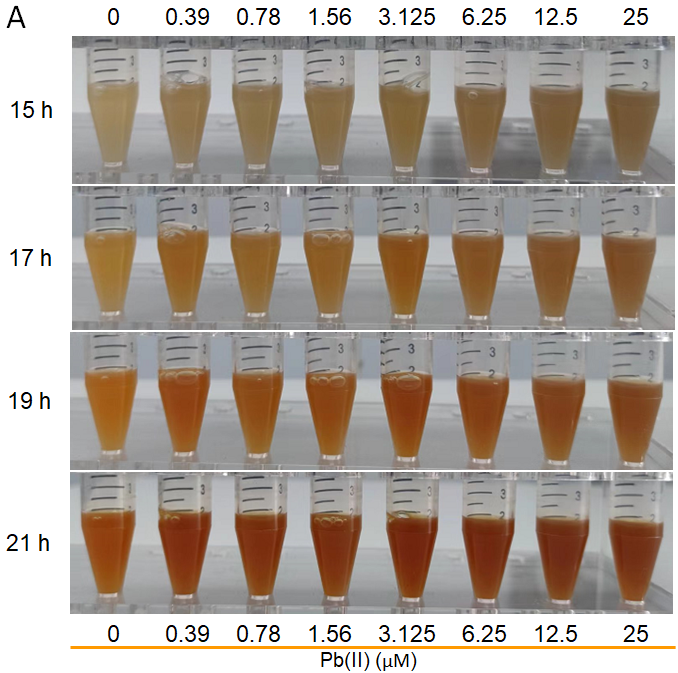

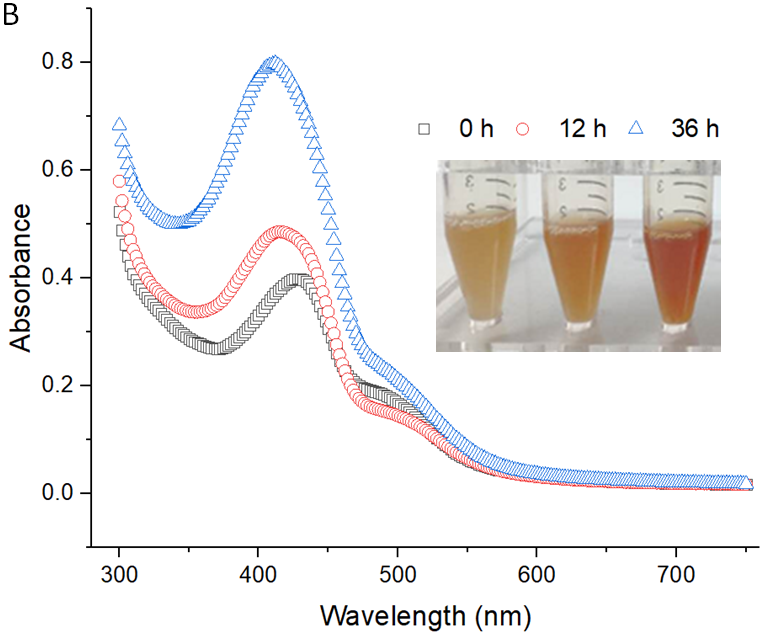


**Fig. S2 The time-dose-response pattern of TOP10/pPb-CACD.**

TOP10/pPb-CACD preserved in 20% glycerol was diluted 1:100 in fresh LB medium supplemented with 1 mM catechin and 50 μg/mL ampicillin. A double dilution method was used to obtain 25, 12.5, 6.25, 3.125, 1.56, 0.78, 0.39, and 0 μM Pb(II) exposure groups, followed by incubation at 37 °C with shaking at 250 rpm. The cultures were sampled at 15, 17, 19, and 21 h (A). The culture upon exposure to 50 μM Pb(II) for 15 h was placed at 37 °C. The cell-free supernatants were prepared and the visible absorption spectrum was scanned at wavelengths of 300-750 nm at intervals of 2 nm at 0, 12, and 36 h (B). The experiments were performed three times and one representative result is shown.


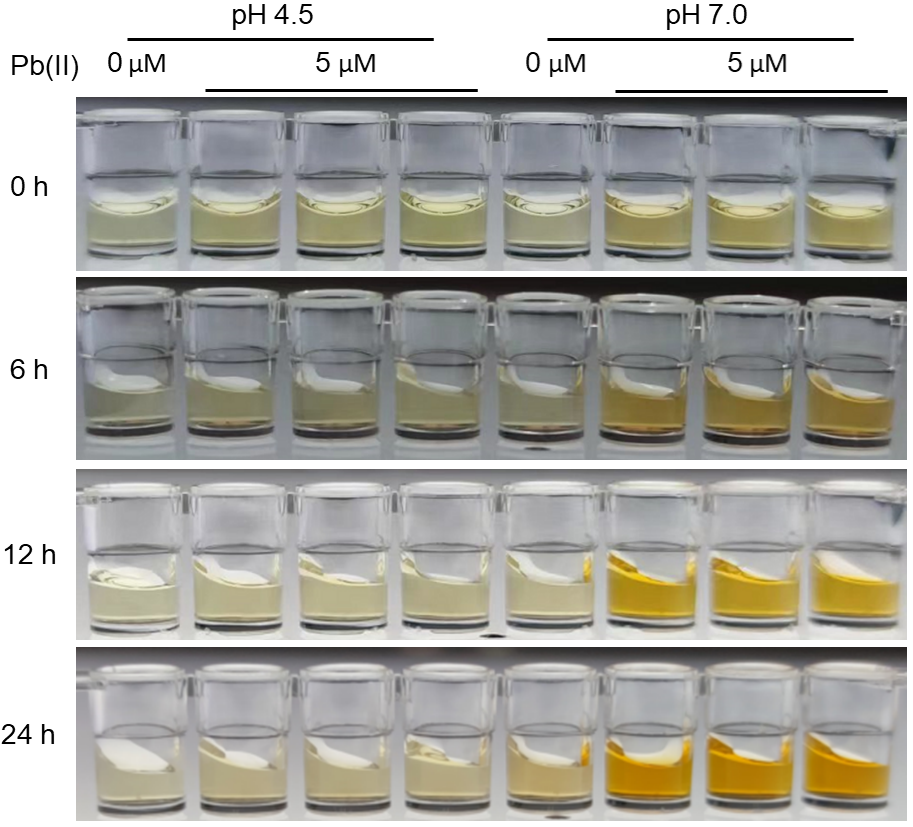


**Fig. S3 The influence of pH value on the stability of pigments in induced culture supernatant.**

TOP10/pPb-CACD was inoculated into fresh LB medium supplemented with 1 mM catechin and induced with 0 or 5 μM Pb(II) at 37 ^o^C for 12 h. The cell-free culture supernatants were prepared and divided into two groups. One group was supplemented with 2% acetic acid (the final pH about 4.5), the other group was the control (the final pH about 7.0), stored at 37 ^o^C and sampled at regular time intervals.

**References**

1. C. Y. Hui, Y. Guo, L. Liu, N. X. Zhang, C. X. Gao, X. Q. Yang and J. Yi, *RSC Advances*, 2020, **10**, 28106-28113.
